# Real-World Evaluation of an AI-Assisted Diagnostic Support System for Early Gastric Cancer: Diagnostic Performance, Confidence Stratification, and Determinants of False-Positive Diagnosis

**DOI:** 10.3390/jcm15072609

**Published:** 2026-03-29

**Authors:** Satoshi Osawa, Takanori Yamada, Wataru Inui, Tomoyuki Niwa, Kenichi Takahashi, Takatoshi Egami, Keisuke Inagaki, Tomohiro Takebe, Tatsuhiro Ito, Satoru Takahashi, Shunya Onoue, Yusuke Asai, Kiichi Sugiura, Tomoharu Matsuura, Natsuki Ishida, Mihoko Yamade, Moriya Iwaizumi, Yasushi Hamaya, Ken Sugimoto

**Affiliations:** 1 Department of Advanced Medical Science for Regional Collaboration, Hamamatsu University School of Medicine, Hamamatsu 431-3192, Japan; 2 Department of Endoscopic and Photodynamic Medicine, Hamamatsu University School of Medicine, Hamamatsu 431-3192, Japan; takanori@hama-med.ac.jp (T.Y.);; 3 First Department of Medicine, Hamamatsu University School of Medicine, Hamamatsu 431-3192, Japan; 4 Department of Laboratory Medicine, Hamamatsu University School of Medicine, Hamamatsu 431-3192, Japan

**Keywords:** artificial intelligence, gastric cancer, upper gastrointestinal endoscopy, false positive, diagnostic confidence, real-world study

## Abstract

**Background/Objectives**: Artificial intelligence (AI)-assisted endoscopy has shown high sensitivity for early gastric cancer detection; however, false-positive diagnoses remain a clinical challenge. This study aimed to evaluate the real-world diagnostic performance of a commercially available AI system and to identify factors associated with false-positive diagnoses, focusing on repeated AI evaluations and confidence stratification. **Methods**: This single-center retrospective study included 47 patients with 89 localized gastric lesions evaluated between March 2024 and March 2025. Endoscopic examinations were performed under white-light, non-magnified observation with repeated AI assessments of each lesion. The rates of “Consider biopsy” (B) judgments were calculated. Lesions with a B judgment rate of ≥50% were defined as AI-positive and classified into four AI confidence categories. Diagnostic performance was assessed using sensitivity, specificity, positive predictive value (PPV), and negative predictive value (NPV). Factors associated with false-positive diagnoses were analyzed using penalized logistic regression. **Results**: The AI system demonstrated a sensitivity of 97.6% and an NPV of 95.7%, with a specificity of 45.8%. Pathology-positive rates decreased stepwise across the four AI confidence categories (*p* < 0.001). Among AI-positive lesions, low regional reproducibility, lesion size ≥ 30 mm, scar, and erosion were independently associated with false-positive diagnoses. In analyses restricted to non-neoplastic lesions, lesion size ≥ 30 mm remained significantly associated with false-positive diagnosis. **Conclusions**: In real-world clinical practice, a commercially available AI system provides high sensitivity for early gastric cancer detection. Incorporating confidence stratification and regional reproducibility into clinical decision-making may enhance the effective use of AI-assisted endoscopic diagnosis beyond binary interpretations.

## 1. Introduction

Upper gastrointestinal endoscopy involving image-enhanced endoscopy and magnified endoscopy plays a central role in the early detection and diagnosis of gastric cancer [1,2]. However, its diagnostic performance remains suboptimal. Previous studies have reported that the false-negative rate for gastric cancer ranges from 4.6% to 25.8%, mainly due to factors such as operator dependency, the small size or subtle morphology of lesions, and variability in observation quality [3,4]. These limitations highlight the need for diagnostic support tools that can improve detection accuracy and reduce interobserver variability.

To address these challenges, artificial intelligence (AI)-assisted diagnostic technologies have been increasingly developed in the field of endoscopic imaging [5,6,7]. The first AI system designed to detect early gastric cancer was reported by Hirasawa et al. [8]. Since then, numerous deep learning–based AI models have been proposed. A recent meta-analysis of 21 studies showed that AI-assisted endoscopy significantly improved the sensitivity of early gastric cancer detection, particularly among non-expert endoscopists [9]. These findings suggest that AI may serve as a valuable adjunct to conventional endoscopy by enhancing diagnostic accuracy.

A newly developed diagnostic support system, gastroAI™ model-G, aims to reduce missed diagnoses and provide high-accuracy endoscopic assessment for early gastric cancer. In a pre-market retrospective performance evaluation, gastroAI™ model-G achieved a sensitivity of 84.7% and a specificity of 58.2%, indicating promising diagnostic capability but also highlighting the issue of a relatively high false-positive rate [10]. In real-world clinical practice, however, several important questions remain unanswered regarding the optimal interpretation and use of AI outputs [11,12]. For example, it should be clarified how to handle situations where the same lesion is repeatedly evaluated and the results are inconsistent, how AI actually works for non-adenocarcinomatous tumors and various benign gastric lesions, and what factors related to the lesion or system contribute to false-positive diagnoses.

The present study aimed to evaluate the diagnostic performance of gastroAI™ model-G (AI Medical Service Inc., Tokyo, Japan) across a broad spectrum of gastric lesions in real-world clinical practice, with particular emphasis on the clinical implications of repeated AI evaluations. In addition, we sought to identify factors associated with false-positive reactions and to clarify whether AI confidence stratification and regional reproducibility could serve as practical indicators for interpreting AI outputs beyond a simple binary classification.

## 2. Materials and Methods

### 2.1. Study Design and Patients

This was a single-center, retrospective study conducted at Hamamatsu University School of Medicine. Consecutive patients who underwent endoscopic examination and/or treatment for suspected early gastric cancer between March 2024 and March 2025 were reviewed.

Patients were considered to have “suspected early gastric cancer” if they were referred to our institution for further evaluation and potential endoscopic treatment following screening endoscopy at other institutions, or if suspicious lesions were identified during screening or surveillance endoscopy at our institution and required further diagnostic assessment. Endoscopic suspicion was based on findings such as irregular mucosal patterns, color changes, or abnormal morphology suggestive of neoplasia, at the discretion of the endoscopist.

This approach reflects real-world clinical practice. In addition, inclusion was partly influenced by the availability of the AI system, which could be used only with a specific endoscopic platform dedicated to detailed examination.

Among these, 47 patients with a total of 89 localized gastric lesions were included in the final analysis.

All lesions were evaluated endoscopically as part of diagnostic assessment or therapeutic procedures for early gastric cancer. The final pathological diagnosis obtained from biopsy or endoscopic resection specimens was used as the reference standard for lesion classification.

### 2.2. Ethics Statement

This study conformed to the principles of the Declaration of Helsinki. In accordance with the Ethical Guidelines for Medical and Health Research Involving Human Subjects issued by the Ministry of Education, Culture, Sports, Science and Technology and the Ministry of Health, Labour and Welfare of Japan, study information, including the objectives, was disclosed on our hospital website with an opt-out approach.

The study protocol was reviewed and approved by the Ethics Committee of Hamamatsu University School of Medicine, Japan (institutional review board approval number: 24-071). Written informed consent was obtained from all participants.

### 2.3. Endoscopic Equipment and Examination Procedure

All endoscopic examinations were performed using the EVIS X1 system (Olympus Medical Systems, Tokyo, Japan) with a GIF-XZ1200 gastroscope. Endoscopic examinations were performed under white-light, non-magnified observation, during which repeated AI assessments were conducted for each target lesion in real time. Video recordings were obtained simultaneously and subsequently used to analyze repeated AI assessments.

### 2.4. AI Assessment and Confidence Categorization

The AI system used in this study was gastroAI™ model-G (AI Medical Service Inc., Tokyo, Japan), a deep learning–based diagnostic support system developed for gastric endoscopic image analysis. All AI evaluations are performed in real time during routine endoscopic examinations. When an endoscopist finds a lesion, they display it on the screen and press the freeze button, which activates the AI system and displays the AI evaluation.

All video recordings were reviewed retrospectively to extract AI judgment data and assess regional reproducibility. This approach enabled a detailed evaluation of both diagnostic performance and the stability of AI outputs under real-world clinical conditions. For each lesion, multiple AI evaluations were performed, and the corresponding AI outputs were documented and recorded. The median number of AI assessments per lesion was five, allowing for analysis of repeated AI outputs and confidence variability.

**Figure 1:**
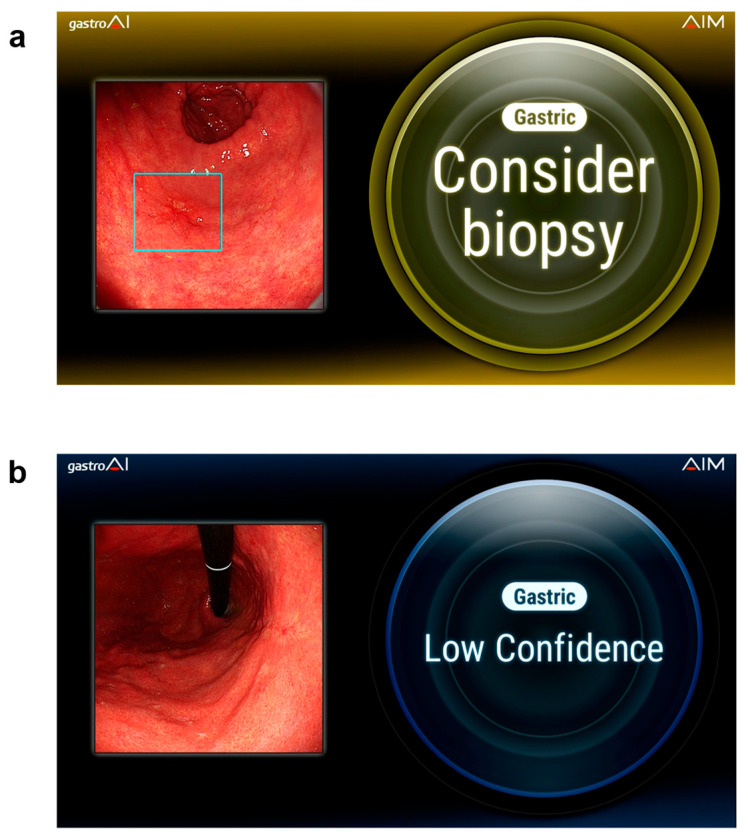
AI assessment by gastroAI™ model-G. The system provides lesion-level outputs indicating the likelihood of neoplastic changes and categorizes findings using “Consider biopsy (B)” and “Low confidence (LC)” judgments. (**a**) The well-differentiated adenocarcinoma in the greater curvature of the antrum was assessed as “Consider biopsy” indicated in a square. (**b**) The benign gastric ulcer scar in the lesser curvature of the middle gastric body was assessed as “Low confidence (LC)”.

For each evaluation, the AI system classified the lesion as either “Consider biopsy (B)” or “Low confidence (LC)” (Figure 1). The B judgment rate for each lesion was calculated as the proportion of evaluations classified as B among all AI assessments performed for that lesion. Lesions with a B judgment rate of ≥50% were defined as AI-positive, whereas those with a B judgment rate of <50% were defined as AI-negative.

Based on the distribution of B and LC judgments across repeated AI assessments, lesions were further classified into four AI confidence categories: B (100% B judgments), B/LC (B judgment rate 50–99%), LC/B (B judgment rate 1–49%), and LC (0% B judgments). These categories were used to evaluate the stepwise diagnostic characteristics and confidence structure of the AI system.

### 2.5. Regional Reproducibility

**Figure 2:**
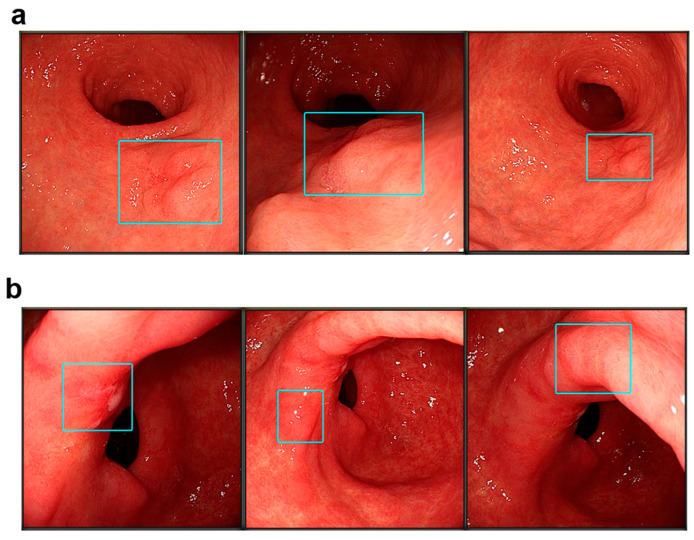
Example of regional reproducibility assessed by AI. Regional reproducibility was assessed to evaluate whether the AI system consistently focused on the same lesion area across repeated evaluations. (**a**) Example of high regional reproducibility (score 1). Repeated AI assessments of gastric adenocarcinoma identified the same area in square, demonstrating regional reproducibility. (**b**) Example without regional reproducibility (score 3). Repeated AI assessment of benign gastric ulcer scars identified discordant areas in square and did not demonstrate regional reproducibility.

Based on the recorded video data, regional reproducibility was assessed to determine whether the AI system consistently focused on the same lesion area across repeated evaluations (Figure 2). Reproducibility was categorized using a predefined scoring system: score 1 (strong reproducibility, ≥70% overlap), score 2 (moderate reproducibility, 50–69% overlap), and score 3 (weak or absent reproducibility, <50% overlap).

This metric was used as an indicator of confidence for AI-positive outputs and was analyzed exclusively among AI-positive lesions, as true-negative lesions are inherently classified as having no reproducible lesion area by definition.

### 2.6. Statistical Analysis

Categorical variables are presented as counts and percentages, and continuous variables are expressed as mean ± standard deviation or median with interquartile range, as appropriate. Differences between groups were assessed using Fisher’s exact test for categorical variables and the Mann–Whitney U test for continuous variables.

Diagnostic performance of the AI system was evaluated using sensitivity, specificity, positive predictive value (PPV), and negative predictive value (NPV), with the final pathological diagnosis as the reference standard.

For analysis of AI confidence, lesions were classified into four categories (B, B/LC, LC/B, and LC) according to the distribution of B and LC judgments. Pathology-positive rates and regional reproducibility scores were compared across the four categories using the χ^2^ test and the Kruskal–Wallis test, respectively. Monotonic trends across increasing AI confidence levels were evaluated using a trend test.

Factors associated with false-positive AI diagnosis were examined in two predefined analyses. First, among AI-positive lesions (biopsy judgment rate ≥ 50%), true-positive and false-positive lesions were compared to identify factors associated with false-positive diagnosis. In this analysis, regional reproducibility was included as a candidate variable because it represents the consistency of AI attention among AI-positive outputs. Second, false-positive lesions were compared with true-negative lesions to identify factors associated with false-positive diagnosis among non-neoplastic lesions; regional reproducibility was excluded from this analysis because true-negative lesions are structurally classified as having no regional reproducibility by definition.

Variables showing potential associations in univariate analyses or considered clinically relevant were entered into multivariable models. Multivariable analyses were performed using penalized logistic regression to account for small sample size and quasi-complete separation. Adjusted odds ratios (ORs) with 95% confidence intervals (CIs) were calculated.

All statistical tests were two-sided, and a *p*-value < 0.05 was considered statistically significant. Statistical analyses were performed using appropriate statistical software.

## 3. Results

### 3.1. Overall Diagnostic Performance of the AI System

A total of 89 gastric lesions were included in the analysis. Baseline characteristics and pathological diagnosis of gastric lesions are presented in Table 1. Based on the predefined threshold for AI positivity (B judgment rate ≥ 50%), the AI system classified 66 lesions as positive and 23 as negative. According to the final pathological diagnosis, there were 40 true-positive, 26 false-positive, 22 true-negative, and 1 false-negative lesions.

The overall diagnostic performance of the AI system showed a high sensitivity of 97.6% and a negative predictive value of 95.7%, whereas specificity and positive predictive value were 45.8% and 60.6%, respectively (Table 2). These results indicate that the AI system rarely missed early gastric cancer but generated a substantial number of false-positive judgments.

### 3.2. Stepwise Diagnostic Characteristics Across Four AI Confidence Categories

Lesions were stratified into four AI confidence categories according to the distribution of B and LC judgments: B, B/LC, LC/B, and LC. As shown in Table 3, the pathological positivity rate decreased stepwise across these categories, with the highest rate observed in the B group (75.6%), followed by the B/LC group (36.0%), the LC/B group (11.1%), and no cancer detected in the LC group (0%). The difference in pathological positivity rates among the four groups was statistically significant (χ^2^ test, *p* < 0.001), and a strong monotonic trend was observed across increasing AI confidence levels (Spearman trend test, *p* < 0.001).

Regional reproducibility scores also demonstrated a significant stepwise deterioration across the four AI confidence categories. It should be noted that lower reproducibility scores indicate stronger regional reproducibility, as score 1 represents high overlap of AI-detected regions. Median reproducibility scores were lowest (indicating the strongest reproducibility) in the B group and progressively increased in the B/LC, LC/B, and LC groups (Kruskal–Wallis test, *p* < 0.001). These findings indicate that the four-tier AI classification reflects a graded confidence structure rather than a simple binary decision.

### 3.3. Factors Associated with False-Positive Diagnosis Among AI-Positive Lesions (TP vs. FP)

To evaluate factors associated with false-positive diagnosis among AI-positive lesions, analyses were restricted to lesions classified as AI-positive (B judgment rate ≥ 50%), consisting of 40 true-positive and 26 false-positive lesions.

**Figure 3:**
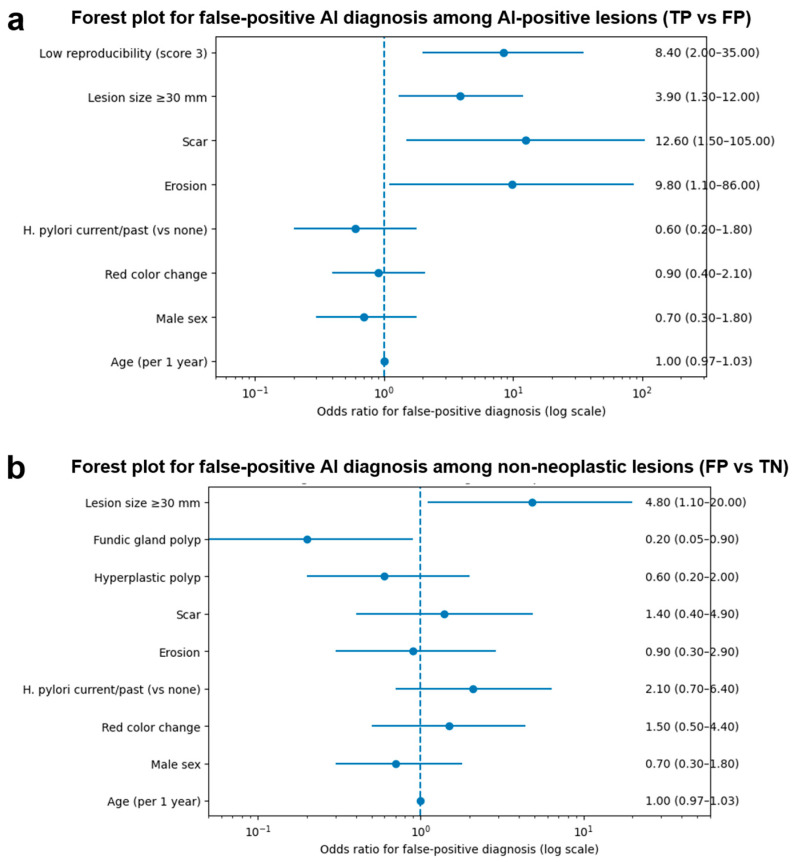
Factors associated with false-positive AI diagnosis. (**a**) Forest plot of univariate odds ratios (ORs) with 95% confidence intervals (CIs) for false-positive AI diagnosis among AI-positive lesions (TP vs. FP). (**b**) Forest plot of univariate odds ratios (ORs) with 95% confidence intervals (CIs) for false-positive AI diagnosis among non-neoplastic lesions (FP vs. TN). Regional reproducibility was excluded from the FP vs. TN analysis due to its structural definition.

In univariate analyses, lower regional reproducibility, larger lesion size (≥30 mm), and the presence of scar or erosion were associated with false-positive diagnosis, whereas age, sex, Helicobacter pylori infection status, and color change were not significant independent predictors (Figure 3a). Multivariable penalized logistic regression demonstrated that low regional reproducibility, lesion size ≥ 30 mm, scar, and erosion were independently associated with false-positive diagnosis among AI-positive lesions (Table 4).

These results indicate that, among AI-positive lesions, regional reproducibility functions as a confidence marker that discriminates true-positive from false-positive diagnoses.

### 3.4. Factors Associated with False-Positive Diagnosis Among Non-Neoplastic Lesions (FP vs. TN)

To assess factors associated with false-positive diagnoses among non-neoplastic lesions, false-positive lesions were compared with true-negative lesions. Regional reproducibility was excluded from this analysis because, by definition, true-negative lesions are structurally classified as having no regional reproducibility.

In this comparison, lesion size ≥ 30 mm was significantly associated with false-positive diagnosis, whereas patient age, sex, Helicobacter pylori infection status, and color change were not significantly associated (Figure 3b). Certain lesion types, such as fundic gland polyps, tended to be less frequently misclassified as false positives, although this association did not consistently reach statistical significance in multivariable analysis (Table 5).

## 4. Discussion

In this real-world, single-center retrospective study, we evaluated the diagnostic performance of an AI-based diagnostic support system for early gastric cancer and investigated factors associated with false-positive AI diagnoses. The principal findings of this study can be summarized as follows: (1) the AI system demonstrated high sensitivity and negative predictive value for early gastric cancer detection in routine clinical practice; (2) repeated AI evaluations revealed a stepwise confidence structure rather than a simple binary output; (3) among AI-positive lesions, false-positive diagnoses were systematically associated with low regional reproducibility and specific lesion characteristics; and (4) among non-neoplastic lesions, large lesion size was a major determinant of false-positive AI reactions. From a methodological perspective, this study provides a real-world framework for evaluating AI-assisted diagnosis using repeated assessments rather than single-image analysis. This approach may be particularly important for clinical implementation, as it reflects the dynamic interaction between endoscopists and AI systems during routine practice.

First, the overall diagnostic performance observed in this study was characterized by high sensitivity with a very low false-negative rate. This finding is consistent with previous reports of AI-assisted endoscopic diagnosis and supports the role of AI as a safety net to reduce missed gastric cancers during routine upper gastrointestinal endoscopy [8,9,13]. Given that missed lesions remain a clinically significant problem even among experienced endoscopists, high sensitivity is a critical prerequisite for AI-assisted screening tools. However, this benefit was accompanied by relatively modest specificity, emphasizing that the clinical value of AI systems cannot be judged by sensitivity alone and that false-positive reactions must be appropriately interpreted and managed.

Second, an important contribution of this study is the demonstration that AI outputs form a graded confidence hierarchy when repeated evaluations are considered. By stratifying lesions into four confidence categories based on the distribution of B and LC judgments, we observed a clear stepwise decrease in pathological positivity rates from the highest- to the lowest-confidence groups. These results indicate that AI-assisted diagnosis should not be interpreted as a binary decision but rather as a probabilistic signal conveying different levels of diagnostic confidence. Such confidence stratification may provide endoscopists with clinically meaningful context when deciding whether to perform a biopsy or continue observation, particularly in borderline or ambiguous lesions [14].

Third, our analysis revealed that false-positive AI diagnoses among AI-positive lesions were not random events but were associated with identifiable factors. Low regional reproducibility emerged as a key discriminator between true-positive and false-positive diagnoses. Regional reproducibility reflects the consistency with which the AI system focuses on the same lesion area across repeated evaluations, and our findings suggest that high reproducibility may serve as a practical confidence marker for AI-positive outputs. In addition, lesion size ≥ 30 mm and the presence of scar or erosion were independently associated with false-positive diagnoses, indicating that extensive mucosal changes and non-neoplastic structural alterations may trigger persistent AI activation despite the absence of malignancy.

Fourth, when false-positive lesions were compared with true-negative lesions among non-neoplastic cases, lesion size ≥ 30 mm remained significantly associated with false-positive diagnosis. This finding suggests that large benign lesions may exhibit morphological features that resemble early gastric cancer and are therefore more likely to be misclassified by AI systems. Conversely, fundic gland polyps tended to be less frequently misclassified as false positives, implying that certain benign lesion types may be inherently less likely to activate cancer-oriented AI algorithms.

These findings should be interpreted in the broader context of current research and clinical implementation of AI-assisted upper gastrointestinal endoscopy. Although numerous studies have reported high diagnostic performance of AI systems for early gastric cancer using image-enhanced or magnifying endoscopy, most of these investigations were conducted under highly controlled conditions, such as the use of static images, preselected datasets, or expert-centered environments [5,15,16,17]. In contrast, AI systems that have obtained regulatory approval and are currently available for routine clinical use remain limited, particularly for gastric cancer detection. This discrepancy highlights a critical gap between experimental AI performance and real-world clinical implementation [12].

**Figure 4:**
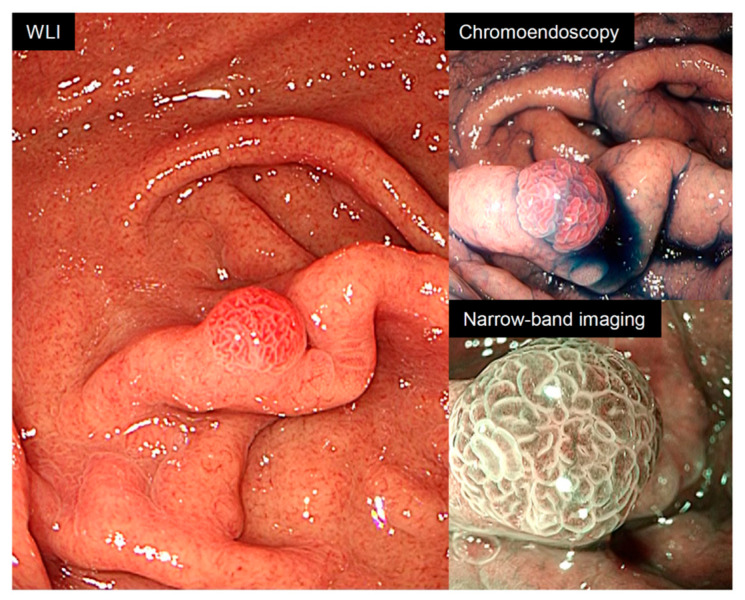
Endoscopic findings of a false-negative case. A small, raspberry-like tumor less than 5 mm in size was observed in the greater curvature of the upper gastric body. White-light imaging revealed a small, reddish, protruding lesion. Chromoendoscopy clearly visualized the lesion surface without irregularity. Narrow-band imaging with magnification revealed irregular microvessels. The lesion was pathologically diagnosed as foveolar-type gastric adenocarcinoma, but the AI system showed a negative result, with a B rate of 33%.

In this study, one false-negative lesion was identified, which was a small raspberry-like tumor (foveolar-type gastric adenocarcinoma) (Figure 4). This lesion presented as a subtle, reddish, protruded polyp and is known to represent a distinct morphological subtype that may be difficult to differentiate from hyperplastic polyps [18]. Notably, this lesion type is not included in the training dataset of the current AI system, as described in the product specifications. Therefore, the false-negative result in this case reflects a predefined limitation of the system rather than a random diagnostic error. This finding highlights an important boundary of AI-assisted diagnosis and underscores the need for continuous model updates to incorporate rare or newly recognized lesion subtypes.

Recent editorials and review articles have emphasized that the clinical acceptability of AI-assisted endoscopy depends not only on sensitivity but also on the interpretability of AI outputs and the management of false-positive detections [15]. In this context, our findings provide practical insight into how AI outputs can be interpreted in routine practice. The observation that false-positive reactions are associated with specific lesion characteristics and AI confidence markers suggests that false positives should not be regarded simply as system errors but rather as signals requiring contextual interpretation by the endoscopist.

Furthermore, recent pre-market studies of updated AI systems (gastroAI™ model-G2) using curated static image datasets have demonstrated the technical evolution of AI platforms under controlled conditions [19]. Although such studies differ fundamentally from real-world, video-based evaluations in terms of target population, dataset composition, and evaluation methodology, they underscore the importance of complementary validation strategies. Together, controlled image-based studies and real-world clinical assessments may provide a more comprehensive understanding of AI performance across different clinical contexts.

From a clinical perspective, the results of this study suggest that future development of AI-assisted upper gastrointestinal endoscopy should move beyond binary classification toward confidence-aware and context-sensitive decision support systems. Incorporating confidence stratification and regional reproducibility into AI outputs may facilitate more appropriate endoscopist–AI interaction, optimize biopsy strategies, and ultimately promote wider clinical adoption of AI technologies in gastric cancer screening and diagnosis.

Several limitations of this study should be acknowledged. First, this was a single-center, retrospective study with a relatively limited sample size, which may restrict the generalizability of the findings. Second, the selection process for “suspected early gastric cancer” may introduce a degree of referral and device-related selection bias, which should be considered when interpreting the generalizability of the results. Third, the number of repeated AI assessments per lesion was determined at the discretion of the examining endoscopist, which may have introduced variability and potential bias in the calculation of AI judgment rates and confidence stratification. Fourth, malignant lymphoma, including mucosa-associated lymphoid tissue lymphoma and diffuse large B-cell lymphoma, was classified as non-adenocarcinoma and analyzed as non-cancer. While this approach allowed a clear focus on early gastric adenocarcinoma detection, it may have influenced the interpretation of false-positive AI reactions for non-epithelial malignant lesions. Fifth, regional reproducibility was assessed using a predefined categorical scoring system based on spatial overlap, and alternative quantitative methods may yield different results. Finally, all examinations were performed using a single endoscopic system at a single institution, and operator- and equipment-specific effects cannot be completely excluded.

In conclusion, this real-world study demonstrates that an AI-based diagnostic support system provides high sensitivity for early gastric cancer detection, while false-positive diagnoses are systematically associated with specific lesion characteristics and AI confidence markers. Integrating confidence stratification and regional reproducibility into clinical decision-making may enable more nuanced interpretation of AI outputs and support the safe and effective implementation of AI-assisted upper gastrointestinal endoscopy beyond binary diagnostic frameworks.

## Figures and Tables

**Table 1:** Baseline characteristics and pathological diagnosis of gastric lesions (n = 89).

Characteristics	Value
Patients, n	47
Lesions, n	89
Age, years (mean ± SD)	71.9 ± 9.5
Male sex, n (%)	74 (83.1%)
Lesion size, mm (median, IQR)	10.0 (5.0–24.0)
Lesion size ≥ 30 mm, n (%)	17 (19.1%)
Lesion location	
Upper third	21 (23.6%)
Middle third	31 (34.8%)
Lower third	35 (39.3%)
Esophagogastric junction	2 (2.2%)
Helicobacter pylori status	
Never infected	18 (20.2%)
Currently infected	15 (16.9%)
Previously infected	53 (59.6%)
Unknown	3 (3.4%)
Pathological diagnosis	
Adenocarcinoma	41 (46.1%)
Malignant lymphoma *	2 (2.2%)
Non-neoplastic lesions	46 (51.7%)
Fundic gland polyp	11
Hyperplastic polyp	8
Ulcer scar	8
Erosion	8
Others ‡	11
Median number of evaluations per lesion	5
Total number of AI judgements	474
Number of considered biopsies (%)	373 (78.9%)
Number of positivity in AI diagnosis (B judgment rate ≥ 50%)	66 (74.2%)

* Malignant lymphoma includes mucosa-associated lymphoid tissue (MALT) lymphoma and diffuse large B-cell lymphoma. ‡ Others include submucosal tumors and other non-epithelial lesions.

**Table 2:** Diagnostic performance of the AI system for early gastric cancer (n = 89).

	AI Positive (n = 66)	AI Negative (n = 23)	Diagnostic Index
Pathology positive (n = 41)	40	1	Sensitivity: 97.6%
Pathology negative (n = 48)	26	22	Specificity: 45.8%
	PPV: 60.6%	NPV: 95.7%	Overall accuracy: 69.7%

AI positivity was defined as a “Consider biopsy” (B) judgment rate ≥ 50%. Sensitivity, specificity, positive predictive value (PPV), and negative predictive value (NPV) were calculated using the final pathological diagnosis as the reference standard.

**Table 3:** Stepwise diagnostic characteristics across four AI confidence categories.

AI Confidence Category	B Judgment Rate (%)	N	True Positive	False Positive	True Negative	False Negative	Pathologically Cancer Positive Rate (%)
B	100	41	31	10	0	0	75.6
B/LC	70–99	25	9	16	0	0	36.0
LC/B	50–69	9	0	0	8	1	11.1
LC	1–49	14	0	0	14	0	0.0

AI positivity was defined as a “Consider biopsy” (B) judgment rate ≥ 50%. Pathology-positive rate differed significantly across the four AI confidence categories (χ^2^ test, *p* < 0.001). The pathology-positive rate in the LC/B group reflects one false-negative lesion. A significant monotonic trend was observed, with decreasing pathology-positive rates from B → B/LC → LC/B → LC (trend test, *p* < 0.001).

**Table 4:** Multivariable analysis of factors associated with false-positive diagnosis among AI-positive lesions (TP vs. FP).

Variable	Adjusted OR	95% CI	*p* Value
Low regional reproducibility (score = 3)	2.70	1.22–6.67	0.015
Lesion size ≥ 30 mm	2.41	1.08–5.38	0.032
Scar	3.68	1.02–13.2	0.047
Erosion	5.43	1.11–26.6	0.037

Multivariable analysis was performed using a penalized logistic regression model to account for quasi-complete separation. Odds ratios (ORs) and 95% confidence intervals (CIs) are shown. Analysis was restricted to AI-positive lesions (biopsy judgment rate ≥ 50%).

**Table 5:** Multivariable analysis of factors associated with false-positive diagnosis among non-neoplastic lesions (FP vs. TN).

Variable	Adjusted OR	95% CI	*p* Value
Lesion size ≥ 30 mm	2.66	1.01–7.03	0.048
Fundic gland polyp	0.59	0.18–1.94	0.39

Multivariable analysis was conducted using a penalized logistic regression model.

## Data Availability

The data presented in this study are available on reasonable request from the corresponding author. The data are not publicly available due to ethical and privacy restrictions related to patient information.

## Abbreviations

The following abbreviations are used in this manuscript:

AI	Artificial intelligence
B	Consider biopsy
LC	Low confidence
NPV	Negative predictive value
PPV	Positive predictive value
OR	Odds ratio
CI	Confidence interval
